# C1QL1 inhibits breast cancer through the HSP90α/VCP-ERS/UPR axis

**DOI:** 10.1038/s12276-025-01486-1

**Published:** 2025-06-30

**Authors:** Ningning Zhang, Qing Shao, Xinni Xiang, Chun Yan, Dan Tao, Qian Li, Huan Rong, Yi Zhao, Tingxiu Xiang, Xiaohua Zeng

**Affiliations:** 1 Department of Breast Cancer Center, Chongqing University Cancer Hospital, Chongqing, China; 2 Chongqing Key Laboratory of Translational Research for Cancer Metastasis and Individualized Treatment, Chongqing University Cancer Hospital, Chongqing, China; 3 West China School of Medicine, Sichuan University, Chengdu, China; 4 Department of Medical Aesthetics, The First Affiliated Hospital of Chongqing Medical University, Chongqing, China; 5 Department of Radiation Oncology, Chongqing University Cancer Hospital, Chongqing, China; 6 Department of Gynecology, The First Affiliated Hospital of Chongqing Medical University, Chongqing, China; 7 Chongqing Key Laboratory for Intelligent Oncology in Breast Cancer (iCQBC), Chongqing University Cancer Hospital, Chongqing, China

**Keywords:** Medical research, Cancer

## Abstract

Our earlier research discovered that C1QL1 was expressed less in breast cancer (BrCa) tissues than in normal breast tissues by analyzing the gene profile of RNA sequences. However, up to now, the biological function of C1QL1 and its molecular mechanism in BrCa remains unknown. Here public database analyses, quantitative PCR with reverse transcription, western blot, immunohistochemistry and quantitative methylation-specific PCR were used to analyze C1QL1 expression and promoter methylation. The effects of C1QL1 on BrCa proliferation, cell cycle, apoptosis and metastasis were assessed using the Cell Counting Kit-8, flow cytometry analysis, terminal deoxynucleotidyl transferase dUTP nick end labeling assays, transwell in vitro and nude mice experiments in vivo. Liquid chromatography–tandem mass spectrometry, co-immunoprecipitation and western blot were performed to identify factors that mediate the effects of C1QL1. In BrCa, C1QL1 is often silenced due to promoter methylation, and its expression is favorably connected with prognosis. Overexpression of C1QL1 inhibits BrCa cell proliferation, metastasis and promotes cancer cell apoptosis both in vitro and in vivo. Conversely, C1QL1 knockdown increases the proliferation and spread of BrCa cells. Mechanistically, C1QL1 is located at endoplasmic reticulum and interacts with HSP90α and VCP to facilitate their ubiquitin-mediated degradation. This leads to the caspase-dependent apoptosis that occurs in BrCa cells as a result of ER stress (ERS)/unfolded protein response (UPR). Our results support that C1QL1 can act as a tumor suppressor of BrCa by modulating the C1QL1/HSP90α/VCP-ERS/UPR pathway, implying that the promoter methylation status of C1QL1 or the expression of C1QL1 may represent a potential marker for the diagnosis or prognosis of BrCa.

## C1QL1 silencing promotes breast cancer progression and metastasis

Breast cancer (BrCa) is a common cancer worldwide, often leading to metastasis and recurrence. This study explores the role of the C1QL1 gene in BrCa. C1QL1 is usually found in the brain but is less expressed in BrCa tissues. This low expression is due to promoter methylation. Here the authors used cell lines and mice to study the effects of C1QL1. They found that increasing C1QL1 levels slowed cancer cell growth and spread by causing cell death through a process called apoptosis. The study also showed that C1QL1 interacts with proteins HSP90α and VCP, leading to their degradation. This interaction triggers stress in the endoplasmic reticulum, an organelle involved in protein folding, which can lead to cell death if not managed. These findings suggest that C1QL1 could be a potential target for new BrCa treatments. This summary was initially drafted using artificial intelligence, then revised and fact-checked by the author.

## Introduction

The incidence of breast cancer (BrCa) is increasing annually and BrCa is one of the most prevalent cancers worldwide^1^. In the carcinogenesis of BrCa, the overexpression of proto-oncogenes coupled with the inactivation of tumor suppressor genes (TSGs) could destroy the balance between cell proliferation and death, by either promoting cell proliferation and/or inhibiting cell apoptosis, resulting in the overgrowth of cells and eventually developing into tumors^2^. Although based on the expression of estrogen receptor, progesterone receptor (PR), human epidermal growth factor receptor 2 (HER2) and Ki67, the molecular classification of BrCa (luminal A, luminal B, HER2 positive and triple negative) has provided considerable benefits^3^, many patients with BrCa experience metastasis or recurrence. Unfortunately, long-term survival statistics for recurrent and metastatic BrCa remain unsatisfactory and treatment remains extremely challenging^4^. Therefore, it is particularly urgent to explore the underlying mechanisms of BrCa and identify new and effective prognostic biomarkers or therapeutic targets for BrCa, which will facilitate the development of effective therapeutic strategies.

C1QL1 (complement component 1, q subcomponent-like 1), as one member of the C1q family/C1QL proteins, was originally cloned as a senescence-related gene and termed C1q-related factor. The C1QL1 gene is located in chromosome 17q21, encoding a protein product composed of 258 amino acids^5^. C1QL1 is highly expressed in the brain and is expressed in different degrees in the testis, uterus, ovary and adipose tissue^6^. Previous studies have found that C1QL1 plays a role in neuronal differentiation, participates in the control of coordinated movement and maintains balance^5,6^. Moreover, C1QL1 was strongly correlated with the activity of Th17 cells and showed a significantly greater level in Th17 cells compared with Th1, Th2 and regulatory T cell subtypes^7^. Furthermore, recent studies suggest that C1QL1 may have potentially important biological functions in tumors. In colorectal cancer, C1QL1 has been identified as a hub upregulated gene involved in various biological processes^8^. In thyroid carcinoma, C1QL1 was highly expressed and positively correlated with tumor size^9^. In glioblastoma, C1QL1 was validated as a new glioma-promoting factor^10^. In addition, in lung adenocarcinoma, C1QL1 is typically upregulated and facilitates tumor cell growth and invasion^11^. Our previous work found that C1QL1 was expressed less in BrCa tissues than in normal breast tissues by examining the gene profile of RNA sequences produced from BrCa and normal breast tissues^12^. Nevertheless, research on the biological role of C1QL1 and its molecular mechanism in BrCa has not yet been conducted.

The endoplasmic reticulum (ER) performs protein secretion and appropriate protein folding to maintain protein homeostasis, which is crucial to determine cell function and fate^13^. However, ER malfunction can result from an accumulation of misfolded proteins in the lumen brought on by a number of different circumstances. This condition is known as ER stress (ERS) and can cause changes in cell phenotypic and related protein expression. It is now recognized that cells initiate the unfolded protein response (UPR), a collection of supplementary adaptive mechanisms that help them deal with changes in protein folding when they experience ERS. To restore equilibrium, the UPR can dynamically modify the ability of the ER to fold and remove improperly folded proteins in response to needs. Additionally, the cytosol and nucleus receive information about the functional state of the ER through the UPR, which causes an upregulation of proteins involved in trafficking, folding, quality control, ER-associated degradation (ERAD) and protein entrance into the ER^14^. However, the UPR can destroy the injured cell by apoptosis if homeostasis of the ER is not adequately maintained^15^. Pancreatic ER kinase (PKR)-like ER kinase (PERK), inositol-requiring enzyme 1 (IRE1α) and activating transcription factor 6 (ATF6), are the three signaling receptors that the ER chaperone glucose-regulated protein 78 (GRP78) dissociates from during the UPR, transducing survival or death signals^13,16^. However, so far, the complex regulatory mechanism of this ERS/UPR is far from clear.

In the current study, we first demonstrated that C1QL1 was downregulated in BrCa partially due to promoter methylation. Ectopic C1QL1 expression inhibited cell proliferation, decreased cell invasion and migration ability, caused cell cycle arrest and induced cell apoptosis in BrCa cell lines. In vivo, C1QL1 decreased BrCa xenograft growth and inhibited experimental lung metastasis. Further mechanistic studies showed that C1QL1 interacted with heat shock protein 90α (HSP90α) and valosin-containing protein (VCP), and downregulated their protein expression via ubiquitin-mediated degradation, resulting in the promotion of ERS/UPR-related caspase-dependent apoptosis. Taken together, our findings provide fresh insight into the regulatory mechanisms underlying the tumor-suppressive actions of C1QL1 in BrCa. The current research may offer an understanding and a theoretical foundation for the creation and implementation of fresh approaches to the detection and management of BrCa.

## Materials and methods

### Cells and reagents

The immortalized human mammary epithelial cell line (MCF-10A), human embryonic kidney cell line (HEK293T) and BrCa cell lines MDA-MB-231, MDA-MB-468, BT-549 and ZR-75-1 were obtained from the American Type Culture Collection. Following conventional procedures, these cell lines were grown in either Dulbecco’s modified Eagle medium or RPMI 1640 medium (Gibco-BRL) supplemented with 10% fetal bovine serum (Gibco-BRL). Cells were cultivated at 37 °C in a humidified environment with 5% CO_2_. MG132, chloroquine (CQ), and tauroursodeoxycholic acid (TUDCA) were purchased from Sigma. KW-2478 and CB5083 were purchased from MedChemExpress.

### Tissue specimens

BrCa and paired adjacent cancer tissue samples were acquired from the Chongqing University Cancer Hospital, Chongqing, China. This study was authorized by Chongqing University Cancer Hospital’s ethics committees and carried out in accordance with the guidelines of the Helsinki Declaration (approval notice CZLS2023295-A). All patients have signed informed consent.

### Bioinformatics analysis

The GENT2 database (http://gent2.appex.kr) was used to analyze the expression status of C1QL1, HSP90α and VCP in BrCa and normal breast tissues. DNA methylation for C1QL1 was analyzed from the DNA Methylation Interactive Visualization Database (DNMIVD)^17^. The data of the important score for each CpG of C1QL1 were acquired from DNMIVD^17^. The Metascape online tool^18^ was used to carry out Kyoto Encyclopedia of Genes and Genomes (KEGG) pathway enrichment analysis and Gene Ontology (GO) functional annotation.

### RNA extraction, RT–PCR and qRT–PCR

TRIzol reagent (Invitrogen) was used to isolate total RNA from cells or tissues in accordance with the manufacturer’s instructions. Promega GoScript reverse transcriptase (Promega) was used to reverse-transcribe aliquots containing 1 μg of total RNA to 20 μl cDNA. The reaction mixture for quantitative PCR with reverse transcription (qRT–PCR) analysis was supplemented with 2 μl cDNA and SYBR green (Invitrogen). The ABI 7500 Real-Time PCR system (Applied Biosystems) was then used to detect the mixture. The qRT–PCR primers are provided in Supplementary Table 1. Each experiment was run in triplicate.

### Aza treatment assay

Cells were collected after applying 10 μmol/l 5‑aza‑2′‑deoxycytidine (Aza) to induce demethylation in cells for 72 h. qRT–PCR was used to determine the mRNA expression level of C1QL1 following RNA isolation. Quantitative methylation-specific PCR was used to measure the methylation and unmethylation levels of the C1QL1 promoter while DNA isolation was completed. The primers for quantitative methylation-specific PCR are provided in Supplementary Table 1.

### Plasmid construction and C1QL1 stably expressing cell lines

C1QL1 expression plasmids (pcDNA3.1-C1QL1-FLAG), full-length and truncated mutant of HSP90α (pcDNA3.1-HSP90α-HA) and VCP (pcDNA3.1-VCP-His) expression were synthesized and purchased from Miaoling Biology. After inserting the entire C1QL1 gene into a pcDNA3.1(+) framework plasmid, transfection was performed using Lipofectamine 2000 (Invitrogen) following the manufacturer’s instructions. To create stable overexpressing C1QL1 cell lines, MDA-MB-231 and MDA-MB-468 were transfected with C1QL1 plasmids and then filtered using G418. Generated control cell lines were transfected using the pcDNA3.1-empty plasmid. Western blot and qRT–PCR were used to verify C1QL1 ectopic expression.

### siRNA

C1QL1 small interfering (si)RNA (siC1QL1) was synthesized by Tsingke Biotechnology Co. Ltd. BT-549 and ZR-75-1 were transfected with siRNA or siNC (control) using Lipofectamine RNAiMAX Transfection Reagent (Thermo Fisher). At 48–72 h after transfection, cells were collected for further tests. C1QL1-siRNA target sequences were as follows: (1) 5′-CGGCAAGUUUACGUGCAACAUTT-3′, 3′-AUGUUGCA CGUAAACUUGCCGTT-5′; (2) 5′-CGAGGUACUCAAGUUUGACGATT-3′, 3′-UCGUCAAACUUGAGUACCUCGTT-5′; (3) 5′-CGGCAAGUUUACGUGCAA CAUCUC-3′, 3′-GAGAUGUUGCACGUAAACUUGCCG-5′.

### Cell proliferation assay

After plating in 96-well plates, cells were allowed to grow for the following 72 h to analyze their capacity for proliferation. Cell proliferation ability was assessed using the Cell Counting Kit-8 (CCK-8). At 0, 24, 48 and 72 h, the OD_450_ value was recorded. Six-well plates were used to plate ectopic C1QL1-expressing or vector-transfected cells (800 cells/well). Surviving colonies (≥50 cells/colony) were counted following fixation and stained with 1% crystal violet after incubation for around 2 weeks. The colonies were photographed with a microscope (Leica DMI4000B) and the Photoshop software was used to count the number of colonies.

### Mobility assays

To evaluate cell mobility, the Transwell assay and scratch wound-healing assay were used. Stable C1QL1-expressing MDA-MB-231 and MDA-MB-468 cells were cultivated in six-well plates until confluent, with the vector-transfected cells serving as controls. Pipette tips were used to properly puncture the cell layers, and phase contrast microscopy (Leica DMI4000B) was used to assess the cell migration distance at different periods. Cell migration and invasion capacity were respectively assessed using Transwell chambers (8 μm pore size; Corning) with or without Matrigel (BD Biosciences). Following fixation and staining, the number of cells on the bottom membrane surface was calculated. Photographs of the migrated cells were taken under a microscope. Each test was run three times.

### Cell cycle and apoptosis experiments using flow cytometry

The cells were taken and preserved in 70% ice-cold ethanol for a whole night to assess the cell cycle. Following this, the cells were rinsed twice with PBS, treated with 5 mg/ml RNase A (Sigma) at 37 °C in the dark for 30 min and then stained with propidium iodide (PI) for 30 min at room temperature. Cells were doubly stained with PI and annexin V-fluorescein isothiocyanate for apoptosis tests. Novoexpress software (Agilent) was used to analyze the data.

### In vivo experiments

Female BALB/c nude mice, weighing between 15 and 19 g and aged between 4 and 6 weeks, were acquired from Vital River Laboratory Animal Technology. Two groups of mice were randomly assigned (*n* = 7 for each group). MDA-MB-231 cells stably expressing C1QL1 and vector (2 × 10^6^ cells in 0.1 ml PBS per mouse) were subcutaneously implanted into the left or right backs of the mice, respectively. Tumors were examined every day. Tumor size was examined every other day and calculated with the formula of 0.5 × length × width × width. After 16 days of injection, the animals were euthanized. The tumors underwent staining tests after being removed, weighed and fixed in paraffin.

Twelve 6-week-old female NOD-SCID mice were randomly divided into two groups (*n* = 6 per group) and given injections of MDA-MB-231-LUC (luciferase transfected) cells that were stable in expressing C1QL1 or vector cells (1 × 10^6^ cells in 0.1 ml PBS per mouse) into their tail veins for metastatic investigations. The IVIS-Lumina Series III (Caliper Life Science) was used for in vivo fluorescence imaging. At 16 days after injection, the fluorescence intensity of the lung was measured in nude mice. For survival analysis, mice were kept for a maximum of 42 days. The ethics committee at Chongqing University Cancer Hospital examined and approved all procedures pertaining to animal care (approval notice CQCH-LAE-20231020008).

### H&E staining

The following was the protocol for hematoxylin and eosin (H&E) staining: tissue slides were deparaffinized using xylene, then hydrated with ethanol for 5 min at concentrations of 100%, 95%, 85% and 75%, then water for 3 min. The procedure involved 3 min of hematoxylin nucleus staining, 5 min of washing with running tap, a brief period of 1% acid ethanol to promote differentiation and a final rinse in running tap water until the cells became blue. Cells were then counterstained for 10 min in 1% eosin. Following dehydration, clearing and mounting, images were taken using an Olympus VS120 slide scanner microscope (Olympus).

### IHC and TUNEL assays

Immunohistochemistry (IHC) staining was carried out in accordance with a previously described procedure^19^ using the following primary antibodies: anti-C1QL1 (Biobyt, orb1260), anti-HSP90α (Abcam, ab2928), anti-VCP (Abcam, ab109240) and anti-PCNA (Santa Cruz, sc-56). Images were photographed on an Olympus VS120 slide scanner microscope (Olympus). Terminal deoxynucleotidyl transferase dUTP nick end labeling (TUNEL) tests were performed using a TUNEL detection kit (Beyotime), and the Olympus IX73 inverted fluorescent microscope (Olympus) was used to record the results.

### IF staining

The pcDNA3.1-vector, pcDNA3.1-C1QL1 was used to transfect the cells after they had been plated on coverslips. As previously described^20^, immunofluorescence (IF) staining was carried out after 48 h. Photomicrographs were acquired with a confocal laser scanning microscope (Leica Stellaris 5). A confocal laser scanning microscope (Leica Stellaris 5) was used to take photomicrographs. IF was conducted using the following antibodies: anti-Flag (1:400, 14793, Cell Signaling Technology), ER-Tracker Red (1:2,000, C1041, Beyotime), anti-HSP90α (1:100, sc-515081, Santa Cruz), anti-VCP (1:100, sc-57492, Santa Cruz), CoraLite 488 (1:200, sc-515081, Proteintech) and CoraLite 594 (1:200, SA00013-4, Proteintech).

### Western blot

Western blot was carried out according to previous methods^20^ using the following primary antibodies: anti-Flag (14793, Cell Signaling Technology (CST)), anti-GAPDH (sc-47724, Santa Cruz), anti-β-actin (sc-47778, Santa Cruz), anti-C1QL1 (orb1260, Biorbyt), anti-Calnexin (2679, CST), anti-HSP90α (8165, CST), anti-VCP (2648, CST), anti-P4HB (3501, CST), anti-ERP57 (2881, CST), anti-GRP94 (2104, CST), anti-GRP78 (3177, CST), anti-PERK (5683, CST), anti-EIF2α (5324, CST), anti-p-EIF2α (3398, CST), anti-ATF4 (ab184909, abcam), anti-ATF6 (65880, CST), anti-IRE1α (3294, CST), anti-CHOP (5554, CST), anti-Bax (2772, CST), anti-Bcl-2 (4223, CST), anti-Caspase 9 (9508, CST), anti-cleaved Caspase 9 (52873, CST), anti-Caspase 7 (12827, CST), anti-cleaved Caspase 7 (8438, CST), anti-Caspase 3 (14220, CST), anti-cleaved Caspase 3 (9664, CST), anti-PARP (9542, CST), anti-cleaved PARP (5625, CST), anti-p62 (8025, CST), anti-LC3α/β (Wl01506, Wanleibio), anti-NLRP3, Gasdermin D (GSDMD, 597558, CST), anti-cleaved Gasdermin D (36425, CST), anti-IL-1β (12703, CST) and anti-ubiquitin (sc-8017, Santa).

### Co-IP

After being lysed in immunoprecipitation-specific RIPA buffer (Absin Bioscience Inc., abs955), specific processed cells were centrifuged at 12,000 *g* for 20 min for co-immunoprecipitation (co-IP). The lysed samples were then incubated for 2 h at 4 °C with protein A and G agarose beads (Absin Bioscience Inc., abs955) to avoid nonspecific binding and then spun. Subsequently, the proteins were incubated with anti-IgG (4 μg, 2729, CST), anti-Flag (1:50, 14793, CST), anti-HSP90α (2 μg, ab2928, Abcam), anti-VCP (1:50, ab109240, Abcam), anti-His (1:100, AE086, ABclonal) or anti-HA (2 μg, 51064-2-AP, proteintech) overnight at 4 °C. After another 2 h of incubation, protein A and G agarose were added. This was followed by a thorough washing with 1× wash buffer. Finally, the precipitated proteins were eluted by boiling in 1× SDS sample buffer and submitted to SDS–PAGE.

### LC–MS/MS assay

Protein samples extracted from stable C1QL1-expressing cells MDA-MB-231 and MDA-MB-468 were incubated with anti-IgG (4 μg, 2729, CST) or anti-Flag (1:50, 14793, CST) and then subjected to the procedures for co-IP. The potential C1QL1-binding proteins were pulled down by co-IP and then liquid chromatography–tandem mass spectrometry (LC–MS/MS) analysis was performed by Wuhan Feiming Biotechnology Co. Ltd.

### CHX, MG132 and CQ assays

To determine the protein half-life, 100 μg/ml cycloheximide (CHX) was applied to the cells, and the cells were then taken for western blot analysis at predetermined intervals. After applying 20 μM MG132 (a proteasome inhibitor) for 8 h or 50 μM CQ (a lysosome inhibitor) for 24 h, the cells were collected for western blotting to analyze the degradation pathway of proteins.

### Statistical analysis

Where appropriate, the Fisher’s exact test, one-way analysis of variance, chi-squared test, Student’s *t*-test or Kaplan–Meier analysis were carried out. For the statistical analysis, GraphPad Prism 8 (GraphPad Software, Inc.) was utilized. The data are presented as mean ± s.d. derived from a minimum of three separate studies. The significance levels of the statistical analyses were *P < 0. 05, **P < 0. 05 and ***P < 0.005.

## Results

### C1QL1 is downregulated due to promoter methylation in BrCa

**Fig. 1:**
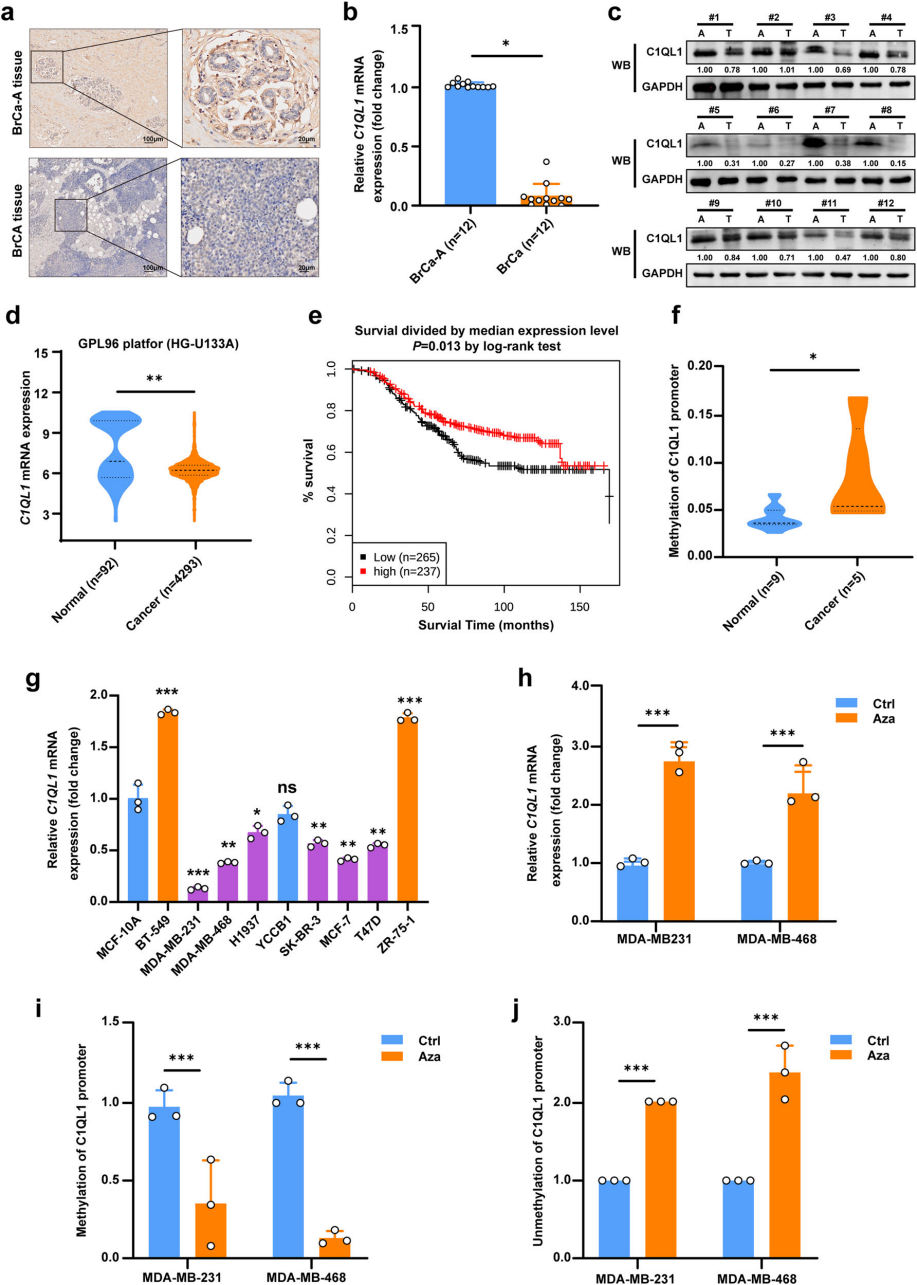
C1QL1 is downregulated due to promoter methylation in BrCa. **a** IHC staining labeled C1QL1 for BrCa adjacent (BrCA-A) and BrCa tissues. **b** The fold change of C1QL1 expression detected by qRT–PCR in BrCa and BrCA-A tissue. **c** C1QL1 protein expression detected by western blot (WB) in BrCa and BrCA-A tissue. **d** The mRNA expression of C1QL1 in breast and normal tissues from the GENT2 database (http://gent2.appex.kr). **e** Analyses of the association between C1QL1 expression and survival in patients with BrCa. Data were obtained from the GENT2 database. **f** Methylation analysis of C1QL1 in BrCa and BrCa-A tissue. **g** qRT–PCR results showing mRNA expression of C1QL1 in breast cell lines. **h** The mRNA expression level of C1QL1 was detected by qRT–PCR after treatment with the demethylating agent Aza compared with control (ctrl). **i**,**j** The methylation status (methylated (**i**) or unmethylated (**j**)) of C1QL1 detected by quantitative methylation-specific PCR after treatment with Aza. All experiments were performed in triplicate. *P < 0.05, **P < 0.005, ***P < 0.0005.

Our earlier research^12^, which used RNA-sequencing screening to identify potential candidate TSGs, revealed that the expression of C1QL1 mRNA was significantly downregulated in BrCa tissues as compared with normal breast tissues. In this investigation, C1QL1 expression was shown to be lower in human BrCa tissues when compared with breast normal tissues, as shown by IHC staining of tissues obtained from our hospital (Fig. 1a). Further analysis using qRT–PCR and western blot revealed that BrCa tissues expressed less C1QL1 than normal tissues (Fig. 1b,c). Based on a larger cohort from the GENT2 database, downregulation of C1QL1 mRNA was consistently seen in BrCa (Fig. 1d). Furthermore, analyzing data from the GENT2 database, we observed that the expression of C1QL1 was lower in HER2-positive BrCa compared with HER2-negative (triple negative and luminal) BrCa tissues. The expression of C1QL1 was lower in PR positive BrCa compared with PR negative BrCa. In addition, the expression of C1QL1 is linked to the grade level and histology subtype of BrCa. However, the estrogen receptor status and differentiation level were not significantly associated with C1QL1 expression (Supplementary Fig. 1a). Further Kaplan–Meier analysis using data from the GENT2 database showed that in patients with BrCa, higher C1QL1 levels were associated with prolonged survival (Fig. 1e).

By analyzing the DNMIVD database, we discovered that methylation of the C1QL1 promoter was substantially greater in BrCa tissues as compared with healthy breast tissues (Supplementary Fig. 1b) and in 23 CpG islands located in the promoter regions of the C1QL1 gene. Supplementary Fig. 1c ranks of importance for these CpGs. Additionally, MethylTarget analysis revealed that, in BrCa tissues as opposed to normal tissues, C1QL1 had significantly greater methylation levels (Fig. 1f). To further identify the correlation between promoter methylation and C1QL1 expression, we used Aza for demethylation on MDA-MB-231 and MDA-MB-468 cell lines, which showed relatively lower expression of C1QL1 than MCF-10A and other BrCa cell lines (Fig. 1g). Figure 1h shows that Aza treatment resulted in the restoration of C1QL1 expression. In these two cells, the methylation status of the promoter decreased and its unmethylation status increased (Fig. 1i, j). Together, these data reveal that promoter methylation might be the explanation for lower C1QL1 expression in BrCa tissues and cells.

### C1QL1 suppresses the proliferation ability of BrCa cells in vitro and in vivo

**Fig. 2:**
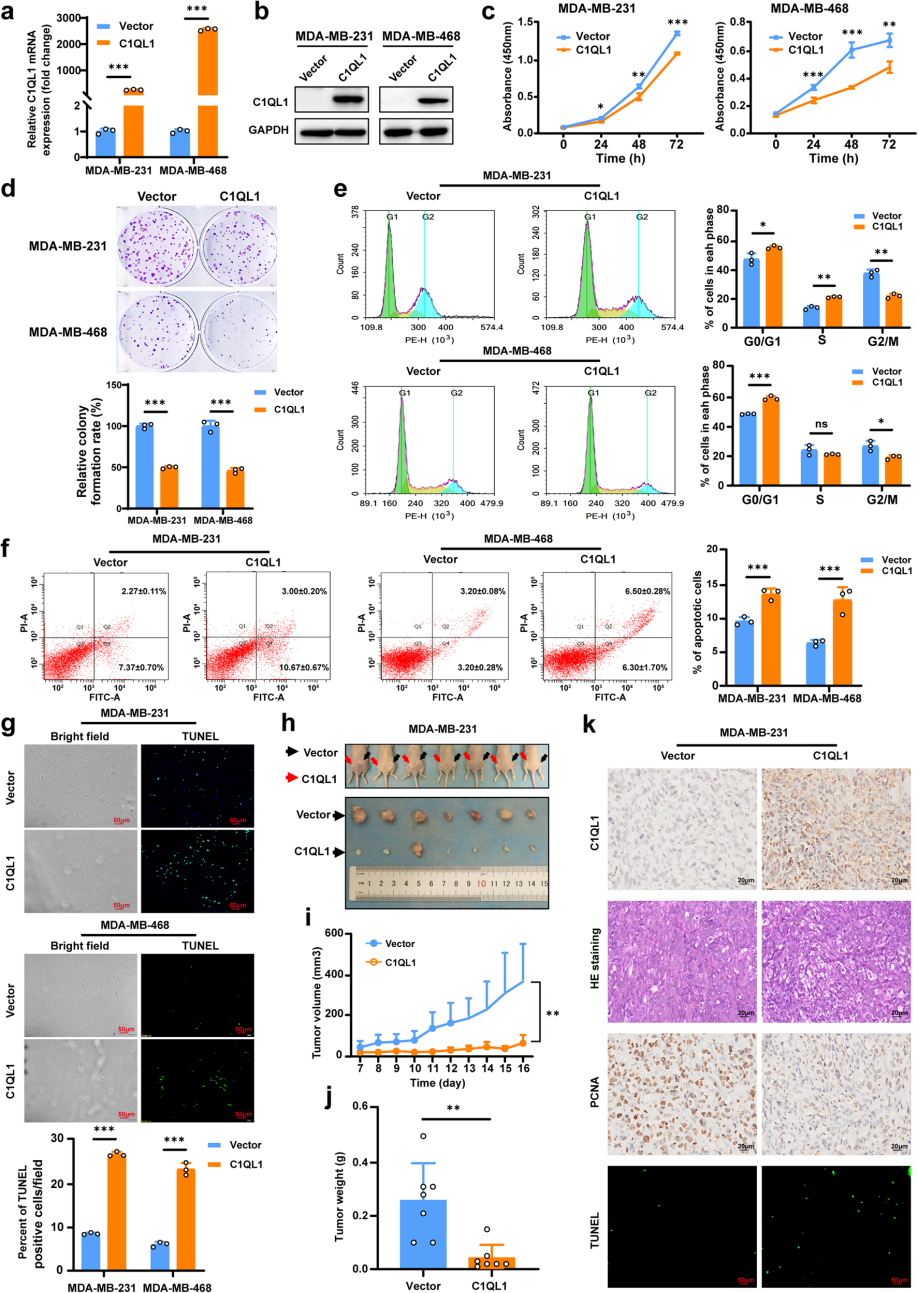
C1QL1 inhibits BrCa cell proliferation in vitro and tumor growth in vivo. **a**, **b** Confirmation of C1QL1-overexpression determined by qRT–PCR (**a**) and western blot (**b**). **c**, **d** Ectopic C1QL1 expression inhibits cell growth, analyzed by a CCK-8 kit (**c**) and colony formation in BrCa cells (**d**). **e**–**g** Ectopically expressed C1QL1 induced cell cycle arrest (**e**) and cell apoptosis tested by flow cytometry analysis (**f**) and a TUNEL assay (**g**). **h** Photographs of the collected tumors. MDA-MB-231 cells stably expressing the vector and Flag-C1QL1 were subcutaneously injected into BALB/c nude mice (*n* = 7). After 16 days of injection, xenograft tumors were collected. **i** The comparative tumor growth curve of the vector and C1QL1 groups. **j** The weights of vector-group and C1QL1-group tumors (*n* = 7). **k** Representative images of C1QL1 expression, H&E staining and PCNA expression. Apoptosis was assessed by TUNEL assays in xenograft tissues. *P < 0.05, **P < 0.05 and ***P < 0.005.

Stable overexpression of C1QL1 was achieved in MDA-MB-231 and MDA-MB-468 cell lines to examine the effect of C1QL1 on the malignant phenotype of BrCa cells (Fig. 2a, b). Assays for CCK-8 cell proliferation and colony formation showed that overexpression of C1QL1 could inhibit the ability of BrCa cells to proliferate (Fig. 2c) and form colonies (Fig. 2d).

Cell cycle distribution experiments were conducted to determine the molecular basis of C1QL1 growth suppression. C1QL1 expression inhibited MDA-MB-231 and MDA-MB-468 in the G0/G1 phase, measured by flow cytometry (Fig. 2e). Additionally, flow cytometry and TUNEL assays demonstrated that C1QL1 markedly elevated the quantity of apoptotic BrCa cells (Fig. 2f, g).

BALB/c nude mice were subcutaneously injected with vector or C1QL1-expressing MDA-MB-231 cells to examine the impact of C1QL1 on the tumorigenic potential of BrCa cells in vivo. Xenograft tumors expressing C1QL1 developed more slowly than those expressing the empty vector, in line with in vitro data (Fig. 2h, i). Similarly, the group that overexpressed C1QL1 had smaller average tumor weights in their xenografts (Fig. 2j). The xenograft tumor features and C1QL1 expression were evaluated using IHC, H&E staining and the TUNEL test. Decreased PCNA staining and increased apoptotic cells were seen in tumor xenografts of cells expressing C1QL1 (Fig. 2k). All of these findings show that BrCa cell proliferation was inhibited by ectopic expression of C1QL1 in both in vitro and in vivo settings.

### C1QL1 inhibits BrCa cell migration, invasion and metastasis in vitro and in vivo

**Fig. 3:**
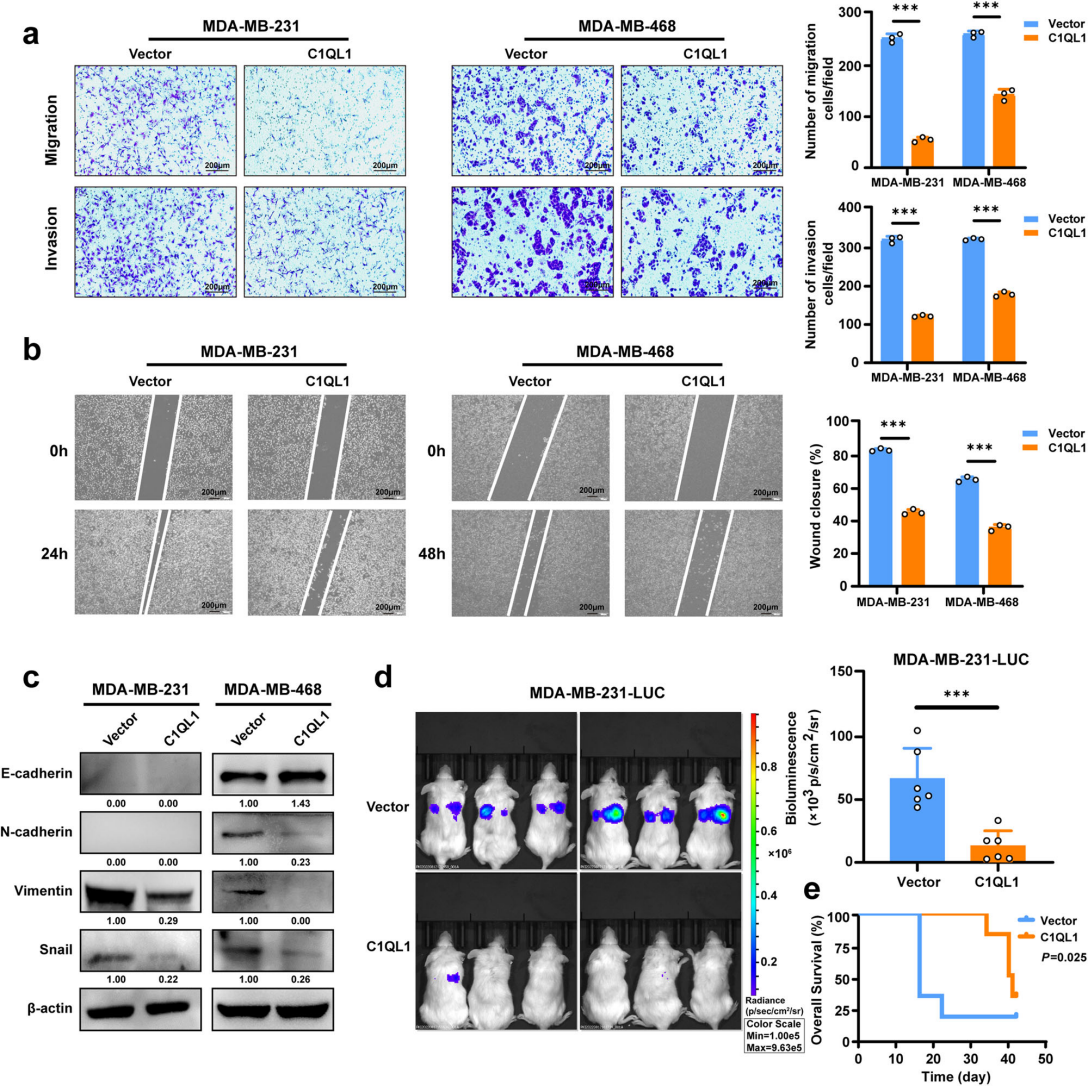
C1QL1 suppresses BrCa cell migration, invasion, EMT and metastasis in vitro and vivo. **a**, **b** MDA-MB-231 and MDA-MB-468 cells stably expressing vector and C1QL1 were subjected to migration assays and Matrigel-coated invasion assays (**a**) and wound-healing assays (**b**). **c** Western blotting analysis of EMT markers in MDA-MB-231 and MDA-MB-468 cells stably expressing vector and C1QL1. **d** Bioluminescence images and intensity of lung metastasis status of NOD-SCID mice at 16 days after vein injection with vector or C1QL1-transfected MDA-MB-231 cells with luciferase activity. Scale bars, 100 μm. **e** Kaplan–Meier curves of survival of mice injected with luciferase-labeled MDA-MB-231 cells stably expressing vector and C1QL1.

We used the Transwell and wound-healing test to further assess the functional effects of C1QL1 on BrCa cells. MDA-MB-231 and MDA-MB-468 cells that were stably expressing C1QL1 demonstrated a markedly reduced ability to invade and migrate, according to Transwell assays (Fig. 3a). The results were verified using the wound-healing experiment, which demonstrated that MDA-MB-231 and MDA-MB-468 cells expressing C1QL1 had a lower wound closure rate than control cells (Fig. 3b). Additionally, we found changes in epithelial–mesenchymal transition (EMT)-associated proteins, which are closely linked to metastasis. Western blotting revealed an increase in E-cadherin (E-cad) expression, along with a decrease in N-cadherin (N-cad), Vimentin and Snail expression in C1QL1 stably expressing MDA-MB-468 cells, while in MDA-MB-231 cells stably expressing C1QL1, the expression of Vimentin and Snail was decreased compared with the vector (Fig. 3c). These results indicate that C1QL1 exerts an inhibitory effect on metastasis by a partial reversal of the EMT process.

MDA-MB-231-LUC (luciferase transfected) cells stably expressing vector or C1QL1 were injected into NOD-SCID mice through the tail vein to investigate whether C1QL1 influences BrCa metastasis in vivo. Using an in vivo imaging system, we saw that mice injected with C1QL1 stably expressing MDA-MB-231-LUC cells showed a significant decrease in luciferase expression 16 days after injection as compared to mice injected with vector control cells (Fig. 3d). Furthermore, compared with mice injected with vector, mice injected with C1QL1 stably expressing MDA-MB-231-LUC cells had a significant survival advantage according to Kaplan–Meier analysis (Fig. 3e). We performed paraffin embedding of lung tissues from the deceased mice, followed by H&E staining. The results revealed the presence of lung metastasis both in the vector and C1QL1 overexpressing groups (Supplementary Fig. 2), which might have contributed to the death of the mice. Taken together, these findings show that C1QL1 may inhibit the spread of BrCa in vitro.

### Knockdown of C1QL1 promotes proliferation, migration and invasion of BrCa cells

**Fig. 4:**
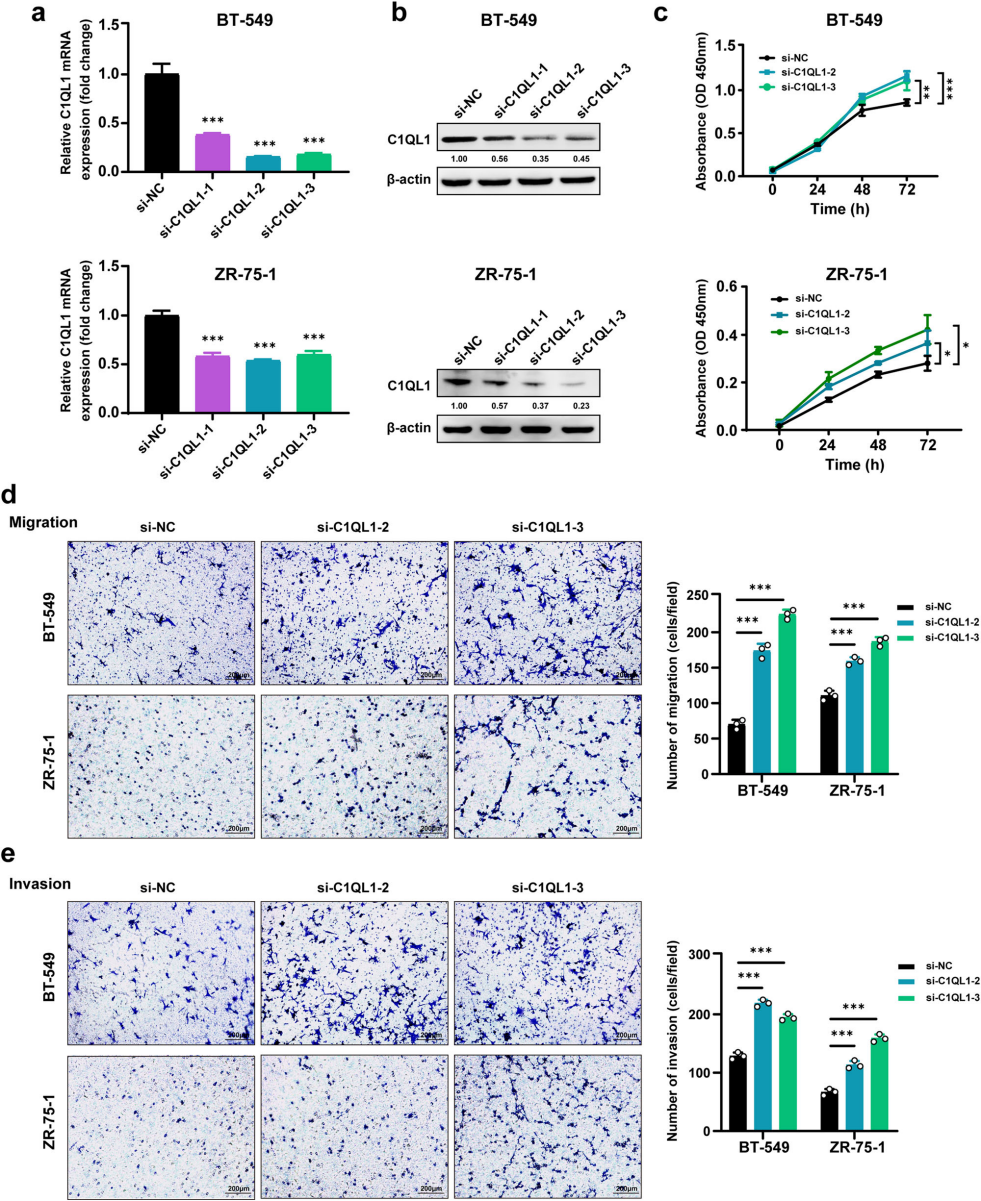
C1QL1 knockdown promotes BrCa cell proliferation, migration and invasion in vitro. **a** C1QL1 mRNA expression was examined by qRT–PCR in BT-549 (top) and ZR-75-1 (bottom) cells transfected with siNC and C1QL1 siRNAs. **b** C1QL1 protein expression was detected with western blot in BT-549 (top) and ZR-75-1 (bottom) cells transfected with siNC and C1QL1 siRNAs. **c** BT-549 (top) and ZR-75-1 (bottom) cells with siNC and C1QL1 siRNAs were subjected to cell proliferation assays using CCK-8. **d**, **e** BT-549 and ZR-75-1 cells with siNC and C1QL1 siRNAs were subjected to migration assays (**d**) and Matrigel-coated invasion assays (**e**).

To evaluate the function of C1QL1, another two BrCa cell lines, BT-549 and ZR-75-1, which had relatively high expression levels of C1QL1 compared with MCF-10A and other BrCa cell lines, were chosen for the knockdown experiments. BT-549 and ZR-75-1 cells were first transfected with siNC (control) or different C1QL1 siRNAs (siC1QL1s), then the C1QL1 expression levels in mRNA and protein were assessed (Fig. 4a, b). SiC1QL1-2 and siC1QL1-3 were selected for functional tests by cell viability and Transwell assays based on knockdown efficiency. The findings showed that C1QL1 knockdown significantly improved cell viability (Fig. 4c) and enhanced the capacity for cell migration and invasion of BT-549 and ZR-75-1 cell lines (Fig. 4d, e).

### C1QL1 interacts with HSP90α and VCP

**Fig. 5:**
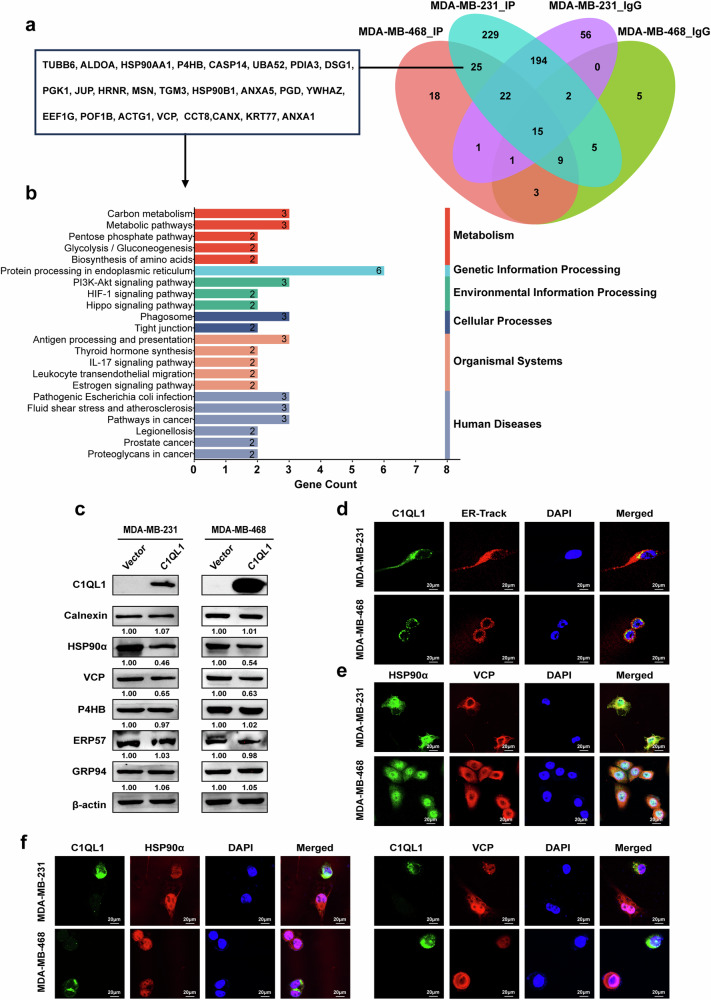
Protein that potentially interact with C1QL1 are mainly enriched in protein processing in the ER and C1QL1 can downregulate the expression of HSP90α and VCP. **a** Co-IP mass spectrometry was used to analyze the proteins that potentially interacted with C1QL1 in MDA-MB-231 and MDA-MB-468 cells stably expressing C1QL1. **b** KEGG enrichment of the genes that encode the 25 proteins that potentially interact with C1QL1. **c** Western blotting analysis of the six proteins enriched in protein processing in the ER in MDA-MB-231 and MDA-MB-468 cells stably expressing the vector and C1QL1. **d**–**f** Immunofluorescence assays were used to detect the protein expression and colocalization of C1QL1 with ER (**d**) HSP90α with VCP (**e**) and C1QL1 with HSP90α and VCP (**f**).

To further determine the molecular mechanism function of C1QL1 in BrCa, LC–MS/MS was used to identify putative proteins interacting with C1QL1 (Fig. 5a). The results showed that 25 proteins might interact with C1QL1. The genes encoding these 25 proteins were analyzed by KEGG pathway enrichment analysis, and 6 genes (CANX, PDIA3, HSP90AA1, VCP, HSP90B1 and P4HB) were found to be enriched in ER protein processing (Fig. 5b). Further detection and analysis showed that C1QL1 could downregulate the expression of HSP90α (HSP90AA1 coding) and VCP at the protein level (Fig. 5c and Supplementary Fig. 3a). To determine whether C1QL1, HSP90α and C1QL1 regulate each other, BT-549 cells were treated with KW-2478 (HSP90α inhibitor) or CB5083 (VCP inhibitor). There was no obvious change in the expression of C1QL1, VCP or HSP90α (Supplementary Fig. 3b,c). In MDA-MB-231 and MDA-MB-468 cells, IF staining showed that C1QL1, HSP90α and VCP partially colocalized with ER (Fig. 5d and Supplementary Fig. 3d), HSP90α partially colocalized with VCP (Fig. 5e) and C1QL1 partially colocalized with HSP90α and VCP (Fig. 5f).

**Fig. 6:**
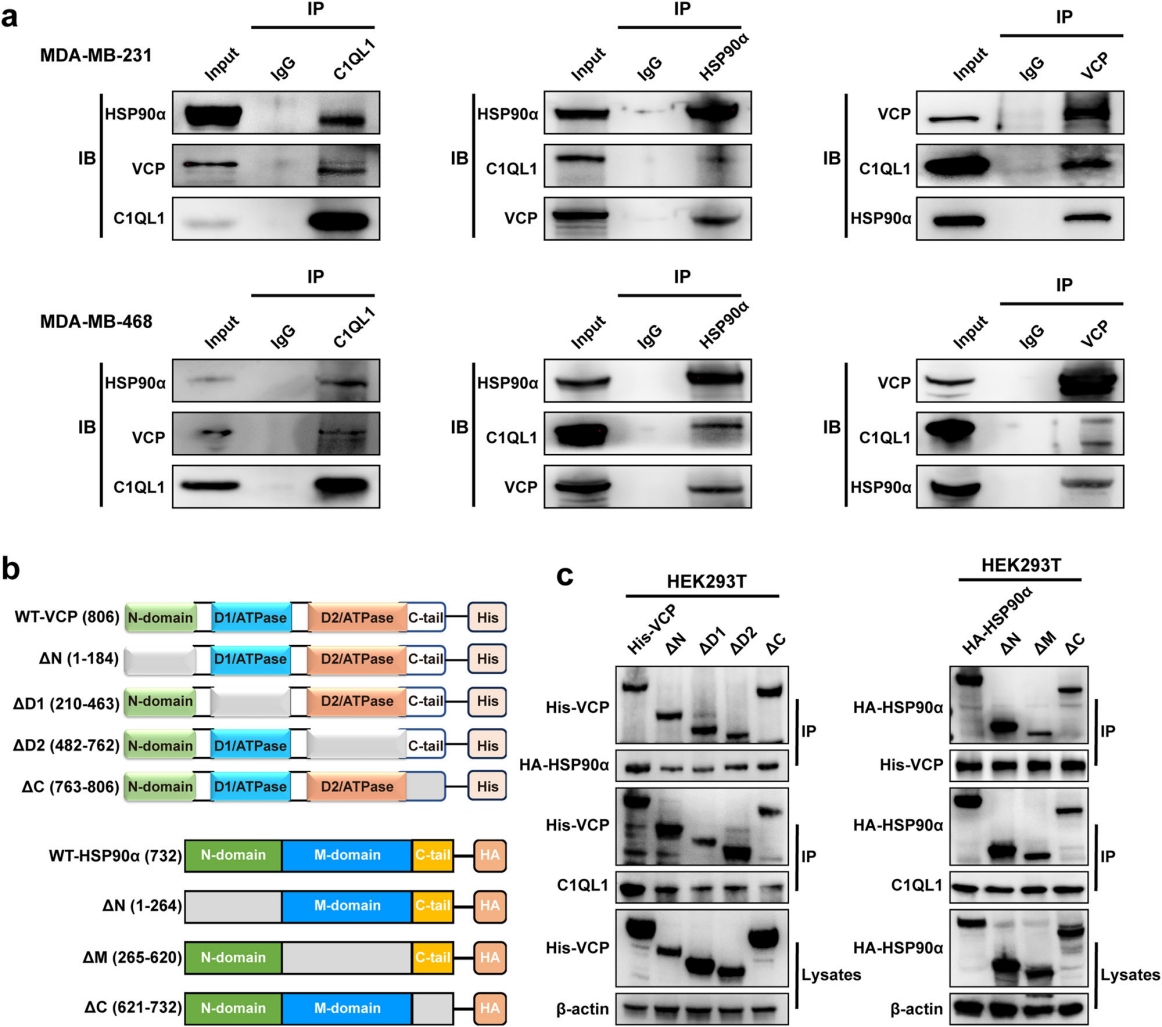
C1QL1 interacts with VCP and HSP90α. **a** The stable expression of C1QL1-Flag in MDA-MB-231 and MDA-MB-468 cells. The indicated proteins were detected by western blot after co-IP with Flag antibodies or HSP90α and VCP antibodies. **b** Top: a schematic for the structural domains of VCP and the N-tail deletion (ΔN, with the deletion of amino acids 1–184), D1 domain deletion (ΔD1, with the deletion of amino acids 210–463), D2 domain deletion (ΔD2, with the deletion of amino acids 482–762) and C-tail deletion (ΔC, with the deletion of amino acids 763–806). Bottom: a schematic for the structural domains of HSP90α and the N-tail deletion (ΔN, with the deletion of amino acids 1–264), M domain deletion (ΔM, with the deletion of amino acids 265–620) and C-tail deletion (ΔC, with the deletion of amino acids 621–732). **c** The interactions of C1QL1, VCP, HSP90α and their mutations were detected in HEK293T cells by co-IP.

To explore whether C1QL1 interacts with HSP90α and VCP, co-IP assays were first performed. The results demonstrated that C1QL1 is bound to endogenous HSP90α and VCP in cells that stably overexpress C1QL1 and vice versa (Fig. 6a). Next, we sought to clarify the essential protein domains underlying the interaction between C1QL1 and HSP90α and VCP. As shown in Fig. 6b, truncated mutation plasmids of HA-tagged HSP90α ((full length (WT), ΔN, ΔM and ΔC)) and His-tagged VCP ((full length (WT), ΔN, ΔD1, ΔD2 and ΔC-tail)) were constructed based on their protein structures. Then, the C1QL1 plasmid or the series of HSP90α and VCP mutant plasmids were transfected into HEK293T cells and co-IP was performed separately. The findings demonstrated that these HSP90α and VCP protein domains interacted with C1QL1 (Fig. 6c).

### C1QL1 attenuates HSP90α and VCP stability and degrades the protein expression of HSP90α and VCP via the ubiquitin–proteasome system

**Fig. 7:**
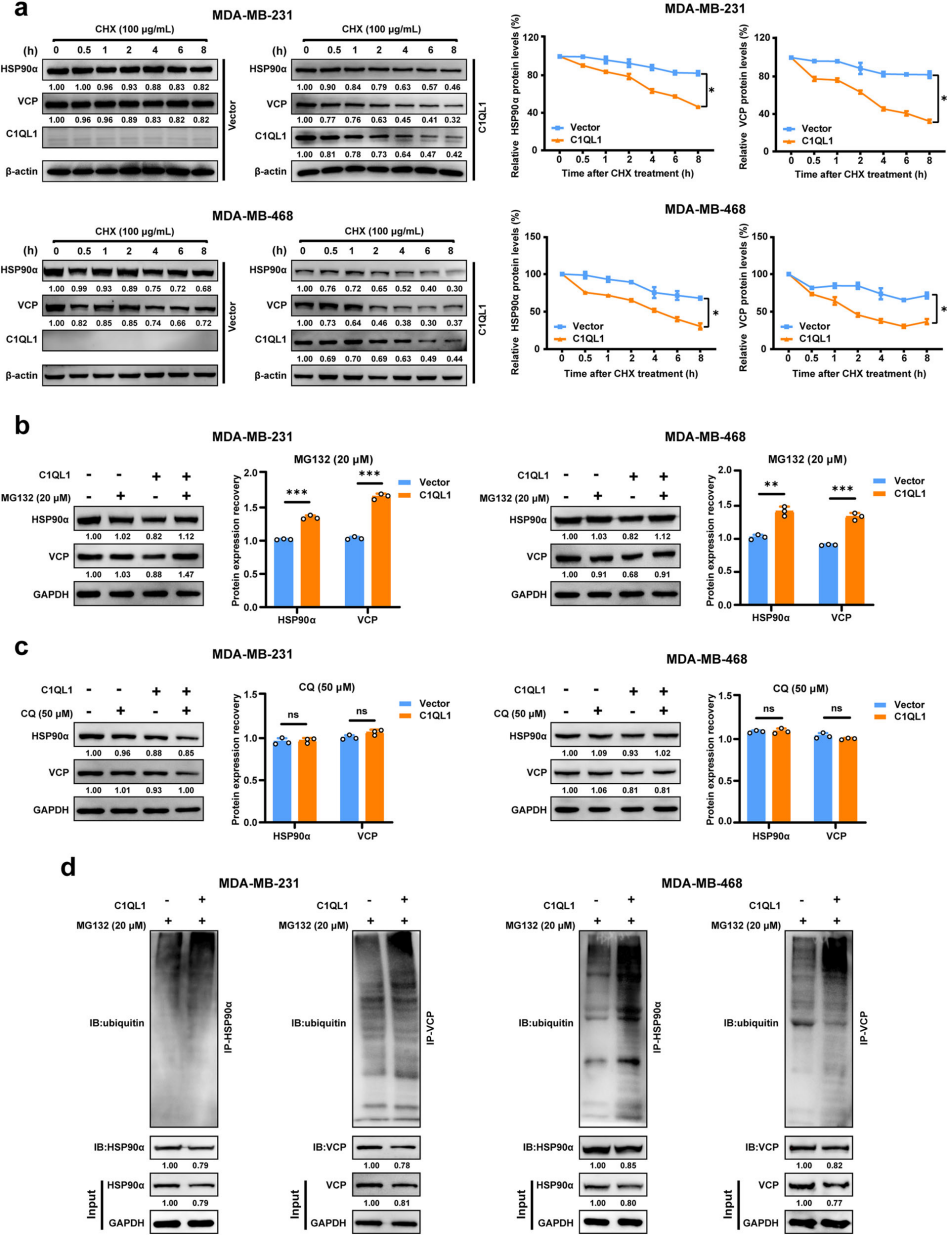
C1QL1 weakens the stability of HSP90a and VCP through the ubiquitin–lysosome pathway. **a** MDA-MB-231 and MDA-MB-468 cells were transfected with vector and C1QL1 plasmids for 48 h, followed by 100 μg/ml CHX treatment for the indicated times. HSP90a and VCP protein expression levels were detected with western blot and quantified with Image J software. Data are presented as the mean ± s.e.m. *P < 0.05. **b** Western blot analysis of the whole cell lysate derived from C1QL1^+/+^ and C1QL1^−/−^ MDA-MB-231 and MDA-MB-468 cells treated with 20 μM MG132 for 8 h. **c** Western blot analysis of the whole cell lysate derived from C1QL1^+/+^ and C1QL1^−/−^MDA-MB-231 and MDA-MB-468 cells treated with 50 μM CQ for 24 h. **d** MDA-MB-231 and MDA-MB-468 cells transfected with vector or Flag-C1QL1 plasmids and treated with 20 μM MG132 for 8 h were immunoprecipitated with HSP90α or VCP, and ubiquitin and protein expression level were detected.

To explore why C1QL1 decreased the expression of HSP90α and VCP, CHX treatment of BrCa cells was first performed. C1QL1 overexpression cells had significantly faster HSP90α and VCP degradation than the control cells (Fig. 7a). The ubiquitin–proteasomal system and the autophagy–lysosomal pathway are the main mechanisms of protein degradation in eukaryotic cells^21^. Thus, the proteasome inhibitor MG132 and lysosome inhibitor CQ were used to treat BrCa cells. The results demonstrated that HSP90α and VCP expression were increased with MG132 but not CQ in the presence of C1QL1 (Fig. 7b, c). Subsequent detection results indicated that MG132 treatment of C1QL1-overexpressed cells resulted in lower protein expression of HSP90α and VCP but elevated ubiquitin levels of these proteins (Fig. 7d). These results showed that C1QL1 overexpression significantly reduced the stability of HSP90α and VCP and increased the ubiquitin levels of HSP90α and VCP.

### HSP90α and VCP are involved in multiple aspects of protein homeostasis

HSP90α is one of the four main subtypes of HSP90, which is a molecular chaperone that participates in protein folding and degradation processes and plays important roles in proteostasis^22–24^. During cellular stress, HSP90 can recognize abnormal or misfolded proteins and assist E3 ubiquitin ligase to realize ubiquitination of these abnormal or misfolded proteins to degrade them by the ubiquitin–proteasome system^25^. HSP90α is related to tumor growth, metastasis and drug resistance^26^, and is regarded as a poor prognostic index in BrCa^27^. VCP is a molecular chaperone belonging to the AAA+ ATPase family. It is attracted to the ER membrane by binding to membrane adapters or its cofactor and is capable of extracting polyubiquitinated proteins from membranes or macromolecular complexes for the proteasome’s subsequent destruction, where it is engaged in ERAD^28,29^. VCP engages in a variety of cellular processes that are essential for cancer cell aggressiveness and survival through interactions with many cofactors. VCP is thought to be a possible cancer biomarker and therapeutic target because it is overexpressed in a variety of cancer types and positively correlates with a bad prognosis^28,30^. Our analyses indicated that both HSP90α and VCP were significantly higher in BrCa tissues than in normal breast tissues and were negatively correlated with prognosis (Supplementary Fig. 4a). Moreover, the GO enrichment results showed that HSP90α and VCP are involved in ERS and UPR processes, including protein folding, the response to ERS, the ubiquitin-dependent ERAD pathway, the ERAD pathway, the ER protein-containing complex, unfolded protein binding, ubiquitin protein ligase binding, ubiquitin-like protein ligase binding and so on (Supplementary Fig. 4b). Ubiquitin detection results further displayed that overexpression of C1QL1 reduced the level of ubiquitination of BrCa cells (Supplementary Fig. 4c).

### C1QL1 regulates the fate of BrCa cells through the ERS/UPR signaling pathway

**Fig. 8:**
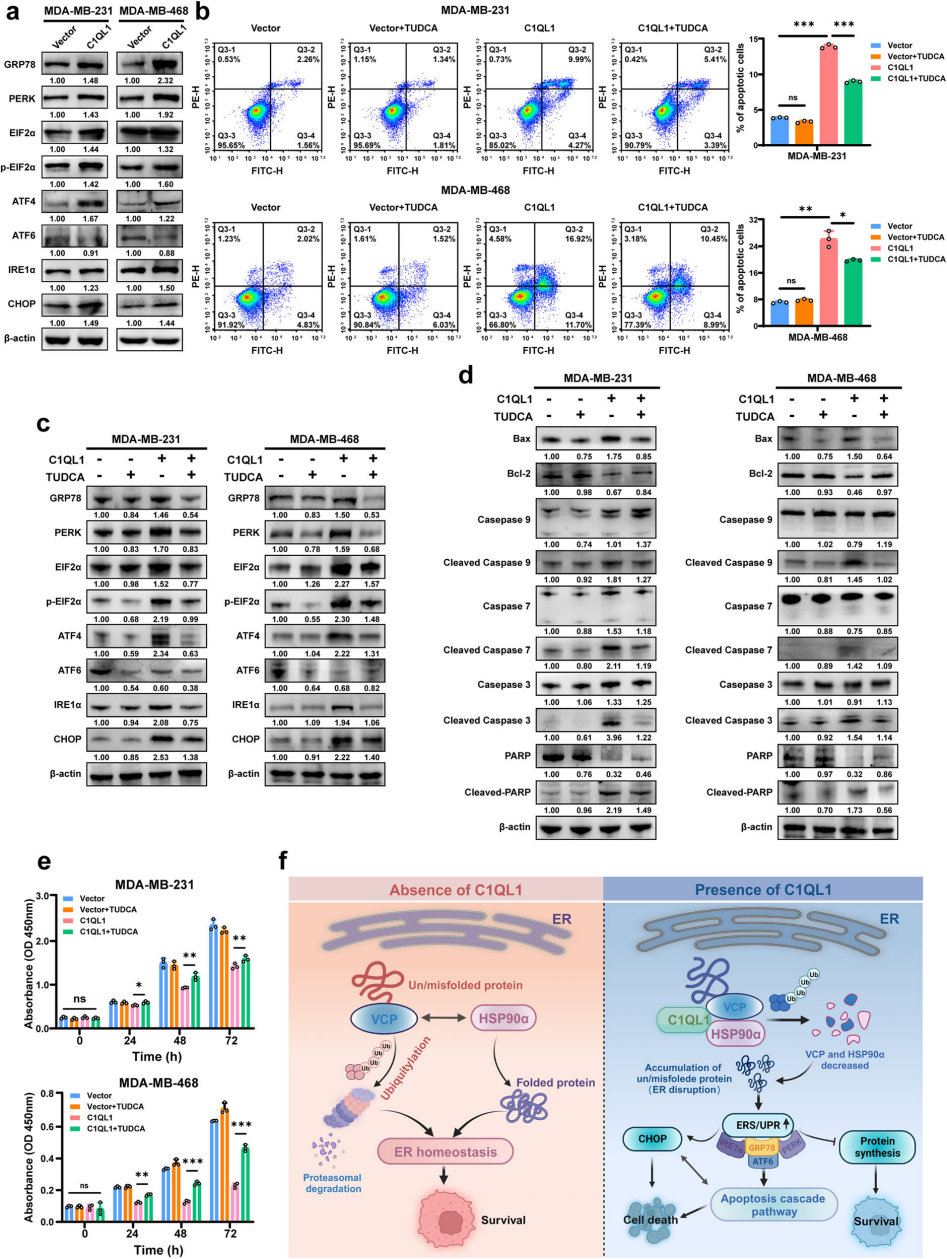
C1QL1 inhibits the growth and induces apoptosis of BrCa cells by ERS/UPR pathway. **a** Markers of ERS/UPR were detected after MDA-MB-231 and MDA-MB-468 cells were transfected with vector and C1QL1 plasmids for 48 h. **b**–**e** MDA-MB-231 and MDA-MB-468 cells were pre-incubated with TUDCA with a concentration of 500 μM for 24 h, then transfected with vector and C1QL1 plasmids for 48 h: cells were collected for flow cytometry analysis of cell apoptosis (**b**) western blot of markers for ERS/UPR pathways (**c**) western blot of ERS/UPR/apoptosis cascade proteins (**d**) and CCK-8 analysis of cell growth (**e**). **f** A schematic outlining the hypothesis of this study.

Our above results suggest that C1QL1 interacts with HSP90α and VCP and reduces their expression, which might result in the accumulation of unfolded proteins or improperly folded proteins in the ER lumen and a state of ERS. Failure to adapt to ERS results in cell death, even though ERS is buffered by the activation of the UPR, a homeostatic signaling network that coordinates the recovery of ER function to preserve the malignant phenotype of tumor cells. Thus we first detected the expression of ERS/UPR-relevant proteins in breast cell lines. As shown in Fig. 8a, ERS-related proteins such as GRP78, PERK, EIF2α, p-EIF2α, ATF4 and IRE1α were upregulated, but ATF6 was downregulated by C1QL1 overexpression. Furthermore, pro-death factors such as CHOP in C1QL1 overexpression cells were significantly higher than in vector cells. Then, we further detected death-related proteins, especially those involved in the core of the executing machinery of apoptosis, pyroptosis and autophagy. The results indicated that Bcl-2 was significantly downregulated, while Bax, cleaved caspase 9, cleaved caspase 7, cleaved caspase 3 and cleaved PARP were significantly upregulated in C1QL1 overexpression cells, while the markers of pyroptosis and autophagy had no obvious expression changes (Supplementary Fig. 5a,b). Flow cytometry showed that the changes in cell apoptosis induced by the upregulation of C1QL1 could be reversed by the ERS inhibitor TUDCA (Fig. 8b). Further western blot results indicated that the changes of the proteins related to the ERS/UPR signaling pathway and apoptosis also be reversed by TUDCA (Fig. 8c, d). As shown in Fig. 8e, pre‑incubation with TUDCA reduced the inhibitory effect of C1QL1 on cell growth. All the results indicated that C1QL1 overexpression could induce ERS/UPR, and modulate protein synthesis and CHOP signaling, further causing caspase-dependent apoptosis, ultimately causing cell growth inhibition.

## Discussion

Abnormal DNA methylation is often closely related to the carcinogenesis of BrCa, which could silence the expression of TSGs and promote cancer development and progression^31^. Several TSGs silenced by DNA methylation have been identified in BrCa by our team, such as DRD2, ZNF334, ZBTB28, ZDHHC22, ZNF474, ZBTB16 and ZMYND10^12,19,20,32–35^. In the current study, C1QL1 was found to be downregulated by promoter hypermethylation in BrCa, and low expression levels of C1QL1 in tumors are significantly associated with unfavorable prognoses. Changes in DNA methylation are known to accompany and contribute to the development of cancer, making them potential cancer biomarkers^36,37^. According to our findings, the expression of C1QL1 or its promoter methylation status may develop into a novel molecular biomarker for the diagnosis or prognosis of BrCa.

Family members of C1QL1 are involved in the metabolism, inflammation and survival signals of various tissue types^38^ and are the initiators of the classical complement pathway^39^. Although previous studies indicated that C1QL1 might have biological functions in malignant tumors^8–11^, the precise role of C1QL1 in BrCa is still unknown, and there is limited research on the molecular mechanism underlying the biological activity of the protein in tumors. This study revealed the important role that C1QL1 plays in preventing BrCa cells from proliferating, migrating, invading and triggering apoptosis both in vivo and in vitro. These results present evidence that C1QL1 is a multifunctional tumor suppressor in BrCa, potentially preventing the malignant functions of BrCa cells both in vivo and in vitro. However, it is worth noting that C1QL1 is frequently upregulated in colorectal cancer^8^, thyroid carcinoma^9^, glioblastoma^10^ and lung adenocarcinoma^11^ and facilitates the invasion and growth of lung cancer cells^11^. It is still unclear what molecular processes underlie the varied functions of C1QL1 in different malignancies. It is probable that C1QL1 regulates different tumors dynamically or that it is determined by diverse interactors in various cancers under varied cellular settings, ultimately contributing to a variety of intracellular activities.

EMT plays a critical role in tumor metastasis^40–42^. E-cad and N-cad are important and the most commonly used biomarkers of EMT. In our study, we were unable to find any evidence of E-cad expression in the MDA-MB-231 cell line. This is in line with our earlier research report^19^ and other studies that claimed MDA-MB-231 was an E-cad-deficient cell line and that E-cad expression was barely detected^43–45^, indicating that MDA-MB-231 is a highly invasive triple-negative BrCa cell line. N-cad as a mesenchymal marker of EMT, is always highly expressed in tumor cells, however, in our study, we failed to detect its expression through multiple experiments. While, other mesenchymal markers of EMT, Vimentin, and Snail could be detected in MDA-MB-231 cells and decreased in C1QL1 overexpressed cells compared with vector cells. Considering the expression of these markers in MDA-MB-468 and the changes following the overexpression of C1QL1, our results indicate that ectopic expression of C1QL1 might prevent BrCa cells from metastasizing by reversing the EMT process. Signaling pathways regulating the EMT process include TGF-β, Wnt-β-catenin, Notch, Hedgehog, PI3K/AKT and NF-κB, which are also involved in various biological processes in tumor cells^46,47^. Thus, we speculate that the antitumor growth effects of C1QL1 not only relate to EMT but also include the regulation of the cell cycle and the induction of apoptosis. Moreover, due to the complex environment of tumors, there is crosstalk between the signaling pathways responsible for different malignant phenotypes. Thus, other forms of cell death, the inhibition of metabolic pathways and the suppression of angiogenesis, which are closely related to the regulation of tumor growth and do not rely on the expression of EMT markers, may also be involved in the C1QL1-mediated growth inhibition of BrCa cells. All these mechanisms warrant further investigation in future studies to achieve a more comprehensive understanding of the broad antitumor effects of C1QL1 in BrCa from multiple perspectives.

In the current study, to determine the underlying mechanisms of C1QL1, IP–mass spectrometry analysis was used to identify the potential interacting proteins. KEGG enrichment analysis of these proteins showed that the most enriched pathway was ‘protein processing in endoplasmic reticulum’. Co-IP and western blot analyses demonstrated that C1QL1 interacted with and enhanced the proteasomal degradation of HSP90α and VCP. IF images further showed that C1QL1 was located on the ER and partly colocalized with HSP90α and VCP.

HSP90α and VCP function as oncoproteins and have been identified as potential therapeutic targets in a number of human tumors^27,28,48^. Both HSP90α and VCP are involved in multiple processes in ERS/UPR, which play an important role in proteostasis protecting the cell from different unfavorable conditions^25,29^. VCP is a co-chaperone of HSP90, which is responsible for maintaining the integrity of ER and is involved in ERAD. In the ERAD progress, VCP cooperates with HSP90 to transport proteins that cannot be refolded or misfolded to the proteasome for degradation, preventing these the aggregation of these proteins, which is the main cause of ERS^49^. Thus, downregulation of HSP90α and VCP may cause abnormal accumulation of unfolded or misfolded proteins in the ER, resulting in ERS/UPR. It is well known that mild ERS can regulate cancer cells to promote cancer cell proliferation, metastasis and drug resistance. Conversely, severe lethal ERS can trigger cell death^13,16^. Our results showed that C1QL1 could induce cell apoptosis and decrease BrCa cell proliferation, invasion and migration capabilities. Overexpression of C1QL1 could alter the expression of key molecules in the ERS/UPR pathway and upregulate ERS-mediated death markers. ERS inhibitors can reverse these molecular changes and restore the growth inhibition and cell death induced by C1QL1. Altogether, our investigations demonstrated that C1QL1 inhibited BrCa through the HSP90α/VCP-ERS/UPR axis (Fig. 8f).

It is worth noting that C1QL1 interacted with all truncated mutation plasmids of both HSP90α and VCP in the current study. This phenomenon may be attributed to the structural and functional characteristics of HSP90α and VCP^23,24,28,29^. HSP90α is likely to possess multiple low-affinity or redundant binding sites, which enable C1QL1 to interact with different regions, even in truncated forms. Furthermore, the conformational flexibility of HSP90α during its ATPase cycle could allow it to adopt alternative structures that facilitate C1QL1 binding, irrespective of truncation. As a molecular chaperone, HSP90α may engage with C1QL1 in a transient, stabilization-driven manner, rather than through sequence-specific interactions. Similarly, the hexameric structure of VCP, which contains multiple AAA+ ATPase domains, offers several redundant binding interfaces, enabling C1QL1 to bind various regions independently. The conformational flexibility and extensive substrate-binding surfaces of VCP may allow for interaction with C1QL1 through general structural features, rather than requiring specific sequence recognition. Additionally, as a chaperone or segregase, VCP may promote nonspecific binding by recognizing substrates in a more generalized fashion. Moreover, hydrophobic or electrostatic interactions could also contribute to binding across multiple truncated forms as these interactions do not depend on a defined binding pocket. Finally, the potential influence of experimental artifacts, such as the overexpression of truncated HSP90α and VCP fragments, may also contribute to nonspecific binding.

It is well known that ubiquitination and deubiquitination, along with their respective enzymes, ubiquitination enzymes and deubiquitinating enzymes (DUBs), are integral processes in cellular protein regulation. Inside the cell, there is a dynamic equilibrium between ubiquitination and deubiquitination, which is crucial for the precise regulation of protein stability and function. These processes regulate each other to ensure that proteins are degraded or stabilized at the correct time. For instance, DUBs can remove ubiquitin chains from proteins that have been incorrectly ubiquitinated, preventing inappropriate degradation^50^. In our study, we found that C1QL1 degrades the protein expression of HSP90α and VCP via the ubiquitin–proteasome system, and this process is certainly mediated by ubiquitination enzymes and DUBs. However, our study did not conduct a more in-depth exploration of the specific mechanisms underlying the ubiquitin-mediated degradation of HSP90α and VCP, such as which specific ubiquitin ligases and DUB are involved in this process. This is a limitation of our study.

C/EBP homologous protein (CHOP), also known as growth-arrest and DNA damage-inducible gene 153 (GADD153), is an important pro-apoptotic factor that is triggered by ERS and is thought to be a sign of commitment of ERS-induced cell death^51^. The expression of CHOP can be induced by the three branches of UPR (PERK, IRE1α and ATF6), especially the PERK–eIF2α–ATF4–CHOP pathway is a dominant apoptotic signaling pathway triggered by prolonged ERS^51^. Our findings demonstrated that C1QL1 overexpression might increase the expression of GRP78, PERK, eIF2α, p-eIF2α, ATF4, IRE1α and CHOP, which suggests that C1QL1 induced ERS/UPR-related cell death mainly through the PERK–eIF2α–ATF4–CHOP pathway and the IRE1α pathway might also contribute to cell death. The core mitochondrial apoptosis pathway, which is controlled by the B cell lymphoma 2 (BCL-2) protein family, is responsible for cell death under ER stress^52^. BAX, as one of the BCL-2 family members, can influence the outer mitochondrial membrane permeability and trigger the caspase cascade, which is a core step in apoptosis^53^. Our results indicated that in C1QL1 overexpression cells, BCL‑2 was downregulated and BAX was upregulated, and the expression of caspase-dependent apoptosis-related proteins, such as cleaved caspase 9, cleaved caspase 7, cleaved caspase 3 and cleaved PARP were upregulated. Moreover, the ERS inhibitor can decrease the apoptotic cascade protein expression induced by C1QL1. These results indicate that C1QL1 partly induced cell death via the ERS/UPR-related caspase-dependent apoptosis pathway in BrCa. Although C1QL1 did not change the expression of makers related to autophagy and pyroptosis, whether other types of cell death, such as necroptosis and ferroptosis, contribute to C1QL1-induced cell death is unknown. Owing to HSP90α and VCP itself functioning as anti-apoptotic factors^28,54^, whether and how they are involved in C1QL1-induced cell death remains unclear. All these questions are worth further studies to determine.

In conclusion, our study first identified C1QL1 as a tumor suppressor in BrCa, which was downregulated by promoter hypermethylation. C1QL1 interacts with HSP90α and VCP and executes a tumor-suppressive function by enhancing the proteasomal degradation of HSP90α and VCP, consequently leading to ERS/UPR-related caspase-dependent apoptosis. These results suggest that the C1QL1 suppressor plays a crucial role in BrCa and may offer a useful diagnostic or prognostic marker for the disease.

## Supplementary information

Supplementary information
