# Supplementary material for: C1QL1 inhibits breast cancer through the HSP90α/VCP-ERS/UPR axis
Source: Exp Mol Med. 2025 Jun 30;57(6):1308–23. doi: 10.1038/s12276-025-01486-1 (PMC12229345; doi:10.1038/s12276-025-01486-1)
Supplement: Supplementary file 1 — Supplementary information [file 12276_2025_1486_MOESM1_ESM.pdf]

Supplementary Fig. 1. The expression and promoter methylation status of C1QL1 in breast cancer. (a) Expression of C1QL1 in different subtypes of breast cancer, data from GENT2 database (http://gent2.appex.kr). (b) Boxplot of DNA methylation for C1QL1 in methylation, data from DNMIVD (http://119.3.41.228/dnmivd/index/). (c) The important score for each CpG of C1QL1, data acquired from the DNMIVD database (http://119.3.41.228/dnmivd/index/) and calculated by XGBoost.

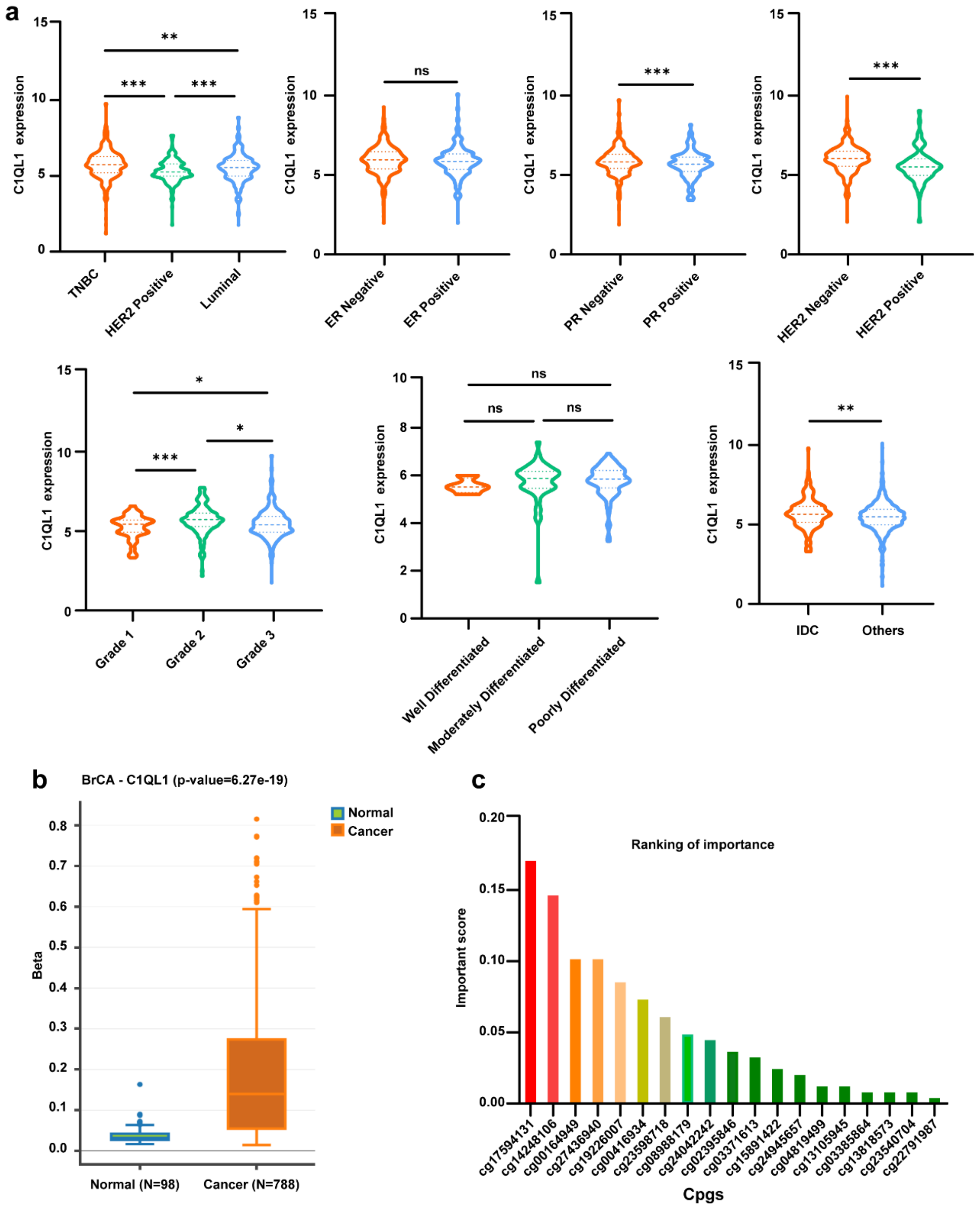

Supplementary Fig. 2. Hematoxylin and eosin (HE) staining of lung metastases in the control and C1QL1 overexpression groups of MDA-MB-231-LUC NOD/SCID mice.

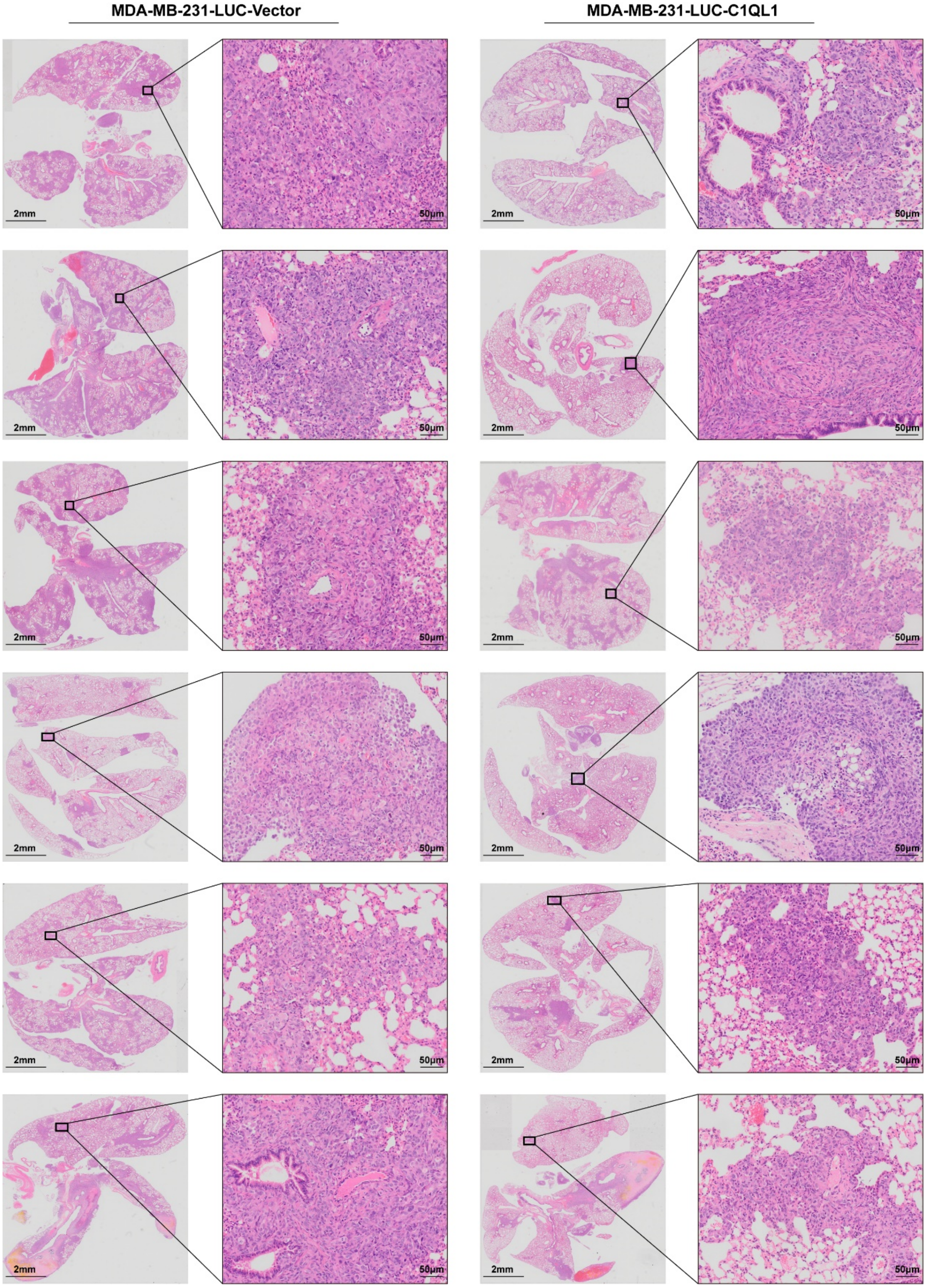

Supplementary Fig. 3. The expression, the association of C1QL1, HSP90α, and VCP, and the co-localization of HSP90α and VCP with ER in breast cancer. (a) C1QL1, HSP90α, and VCP mRNA expression were examined with qRT-PCR in MDA-MB-231 and MDA-MB-468 cells expressed with vector and C1QL1. (b) C1QL1, HSP90α, and VCP protein expression were detected by western blotting in BT-549 cells after 24h of exposure to different concentrations of HSP90α inhibitor KW-2478. (c) C1QL1, HSP90α, and VCP protein expression were detected by western blotting in BT-549 cells after 8h of exposure to different concentrations of VCP inhibitor CB5083. (d) Immunofluorescence assays were used to detect the co-localization of HSP90α and VCP with endoplasmic reticulum.

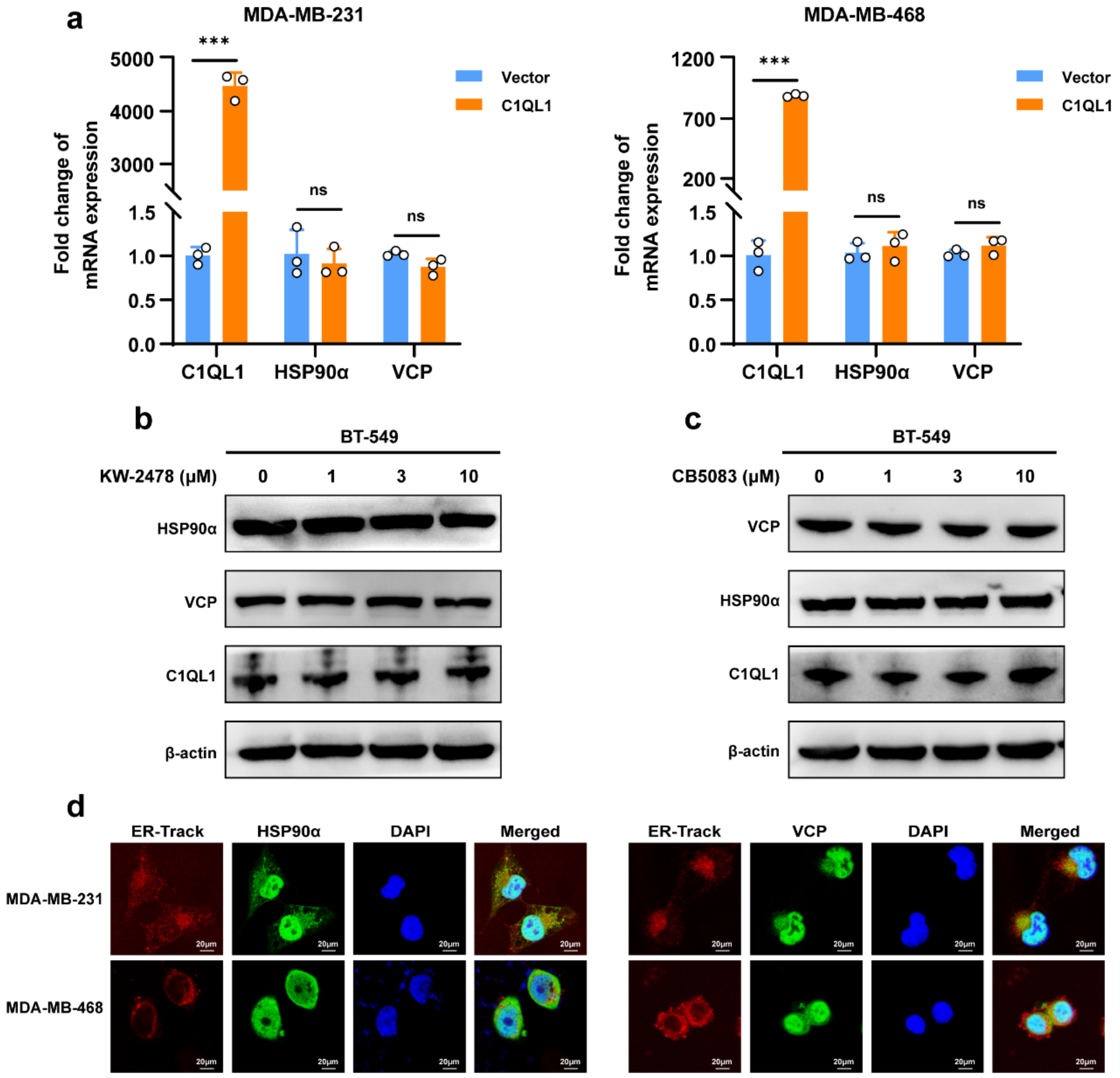

Supplementary Fig. 4. The expression and biological function analyses of HSP90α and VCP in breast cancer. (a) The mRNA expression of HSPAA1, VCP, and their association with overall survival in breast cancer. Data were obtained from the GENT2 database (http://gent2.appex.kr). (b) GO function enrichment of HSP90α and VCP using Metascape (https://metascape.org). (c) MDA-MB-231 and MDA-MB-468 cells transfected with vector or Flag-C1QL1 plasmids for 48 hours, ubiquitin and expression level of HSP90α and VCP were detected with western blot.

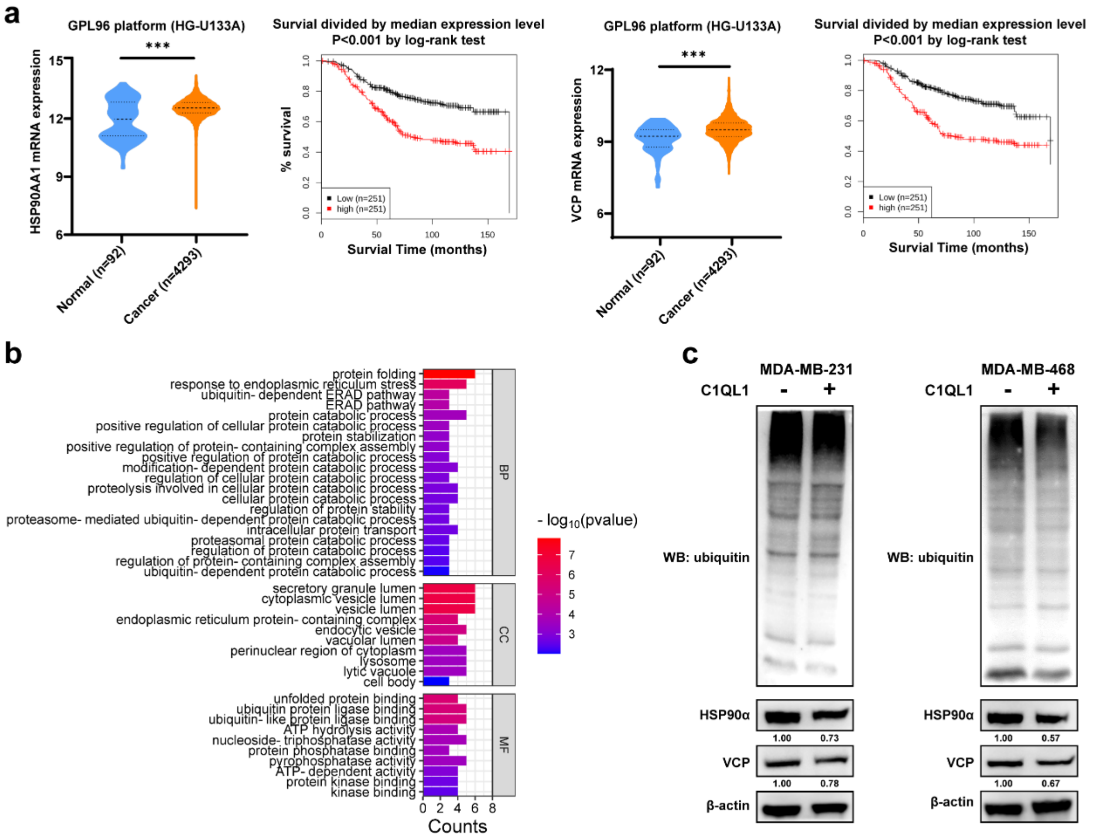

Supplementary Fig. 5. Apoptosis-associated proteins are regulated by C1QL1. MDA-MB-231 and MDA-MB-468 cells were transfected with vector and Flag-C1QL1 plasmids for 48 hours, protein were harvested for western blot. (a) Apoptosis cascade proteins were detected. (b) Pyroptosis and autophagy-related proteins were assessed.

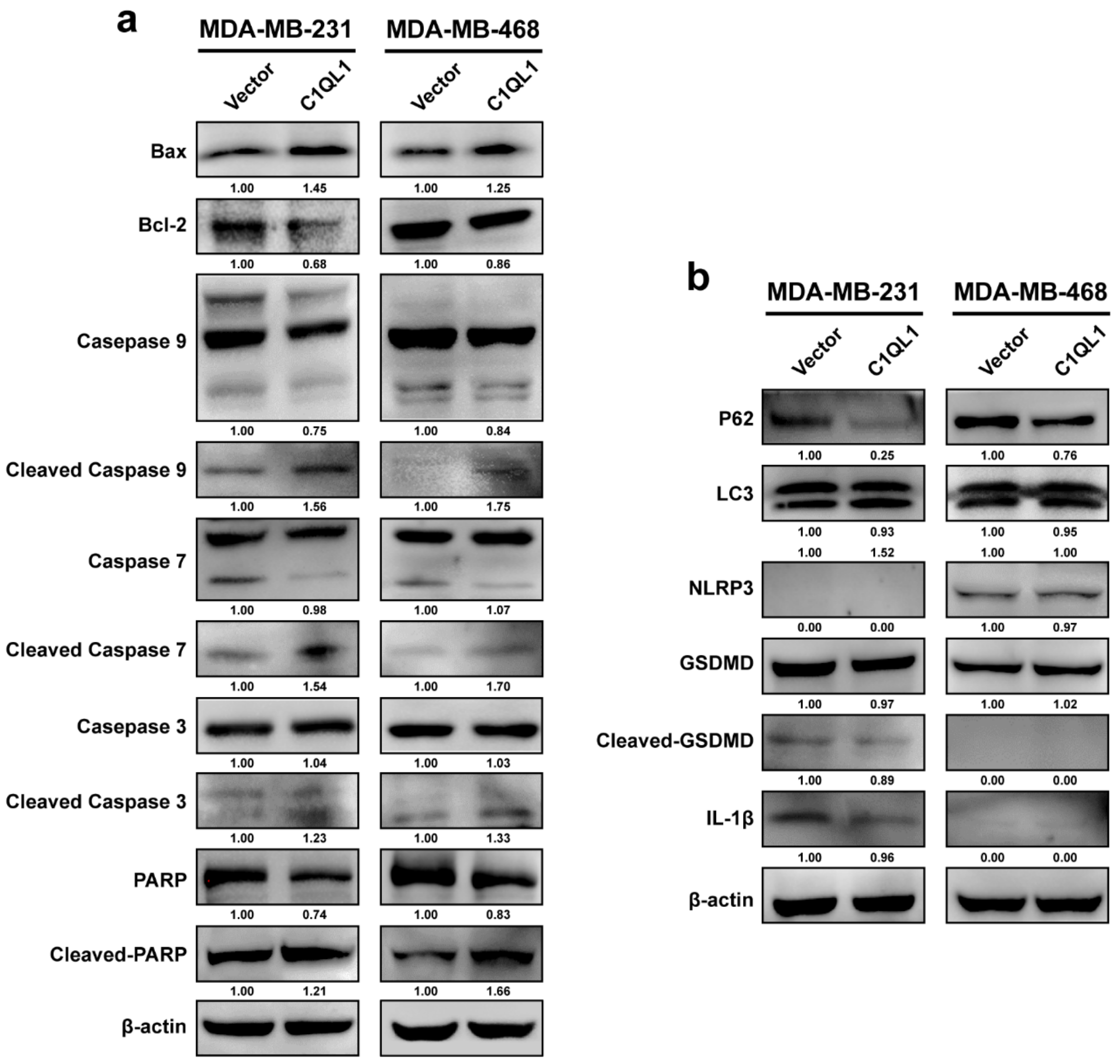

Supplementary Table 1. List of primers used in this study.

PCR	Primer	Sequence	Product size (bp)	PCR cycles	Annealing temperature (°C)
qRT-PCR	C1QL1F	GGGTTACGAGGTACTCAAGTTTG	103		60
	C1QL1R	AAAGTAGGTGCCGGGAATGTT			
	HSP90AA1F	AGGAGGTTGAGACGTTCGC	223		60
	HSP90AA1R	AGAGTTCGATCTTGTTTGTTCGG			
	VCPF	AGGAGCCAGCGTTGTTCGCC	154		60
	VCPR	GCGGGTAACGGCTACGAGCG			
	β-actinF	CATGTACGTTGCTATCCAGGC	250		60
	β-actinR	CTCCTTAATGTCACGCACGAT			
MSP	C1QL1m1	GCGCGGGCGTTTAGTTCGC	133	40	60
	C1QL1m2	CATCACCACACCCGCGACG			
	C1QL1u1	GAGTGTGGGTGTTTAGTTTGT	135	40	58
	C1QL1u2	ACATCACCACACCCACAACA			
